# Procurement architecture as a determinant of rehabilitation system performance: insights from Ukraine's prosthetics sector

**DOI:** 10.3389/fresc.2026.1870568

**Published:** 2026-06-29

**Authors:** Aadrian Sullivan, Volodymyr Golyk, Marlee Elizabeth Quinn, Oleh Karpov

**Affiliations:** 1 Consultant, Copenhagen, Denmark; 2 Rehabilitation and Disability Unit, WHO Country Office in Ukraine, Kyiv, Ukraine

**Keywords:** assistive technology, procurement, prosthetics and orthotics, rehabilitation systems, Ukraine

## Abstract

Access to assistive products remains highly uneven across health systems. Policy discussions in rehabilitation frequently emphasize workforce development, service delivery models, and clinical standards. Less attention has been given to the institutional mechanisms through which assistive products are procured and supplied. Ukraine's prosthetics sector provides a contemporary illustration of how procurement systems influence rehabilitation service delivery. Following the escalation of armed conflict in 2022, Ukraine experienced a sharp increase in demand for prosthetic services. National programmes expanded rapidly to support individuals requiring rehabilitation and assistive technologies. In this context, procurement arrangements - including decentralized purchasing across multiple providers and reliance on imported prosthetic components, have highlighted structural challenges affecting supply chain coordination, pricing transparency, and system scalability. This paper argues that procurement architecture represents an under-recognized determinant of rehabilitation system performance. Drawing on insights from Ukraine's prosthetics sector, the paper explores how fragmented procurement arrangements can reduce cost efficiency in access to assistive technologies even when financing and service delivery capacity expand. It further outlines procurement reform principles that may strengthen rehabilitation systems, including pooled purchasing mechanisms, framework agreements, demand forecasting integration, and digital procurement platforms.

## 1 Introduction

Rehabilitation services are increasingly recognized as an essential component of health systems (1). Global estimates suggest that more than 2.4 billion people worldwide could benefit from rehabilitation interventions, reflecting the combined effects of population ageing, rising prevalence of chronic disease, and increased survival following injury and trauma. International health policy frameworks have emphasized the importance of strengthening rehabilitation systems as part of broader efforts to advance universal health coverage and improve population health outcomes (2).

Initiatives such as Rehabilitation 2030 highlight the need for governments to integrate rehabilitation services more fully into national health systems. This includes expanding rehabilitation workforces, strengthening service delivery networks, and ensuring access to essential assistive technologies. Assistive technology - including prosthetic and orthotic devices - play a critical role in enabling individuals with functional limitations to regain mobility, independence, and social participation (3).

Despite growing recognition of the importance of rehabilitation services, access to assistive technologies remains limited in many contexts. Global assessments indicate that a substantial proportion of individuals who require assistive products are unable to obtain them due to financial barriers, limited-service capacity, or supply chain constraints (4). But equally, how you buy is as important as what you buy (5), as strategic purchasing can contribute and assist in advancing universal healthcare coverage. While rehabilitation policy discussions frequently focus on clinical service delivery and workforce development, comparatively less attention has been given to the institutional systems that determine how assistive technologies are purchased and distributed (6).

Procurement systems represent a critical element of this institutional infrastructure. Procurement encompasses the policies, processes, and governance arrangements through which medical devices are acquired within health systems (7). In the context of rehabilitation services, procurement determines how prosthetic components, orthotic materials, and other specialized assistive technologies are sourced from domestic and international suppliers (8).

Although procurement is often treated primarily as an administrative function, its structure has significant implications for the availability and affordability of assistive technologies (9). Procurement arrangements shape supplier markets, influence pricing, and affect the reliability of supply chains. When procurement systems function effectively, they enable rehabilitation providers to access the components required for patient care in a timely and cost-effective manner (10). Conversely, fragmented or poorly coordinated procurement arrangements may create barriers to accessing essential assistive technologies (3, 11).

Ukraine's prosthetics sector provides a valuable illustration of these dynamics. Following the escalation of armed conflict in 2022, the country experienced a substantial increase in demand for prosthetic services among both civilian and military populations (12). National authorities responded by expanding public programmes designed to provide prosthetic devices and other rehabilitation aids to individuals requiring support (13). These efforts resulted in tens of thousands of individuals receiving assistive technologies through state-supported programmes providing in the first 6 months of 2025, over 56 000 people with 208 000 assistive products (14).

As prosthetic services expanded rapidly, increasing by 80% from an estimated 50 registered service providers in 2022 to 93 registered service providers in 2025, procurement arrangements for prosthetic components emerged as a key factor shaping the capacity of providers to deliver care (15, 31). The Ukrainian experience demonstrates how procurement architecture influences the ability of rehabilitation systems to respond effectively to increased demand.

## 2 Prosthetics provision and procurement dynamics in Ukraine

Prosthetic services involve the design, fitting, and maintenance of devices that replace or support missing or impaired limbs (16). Modern prosthetic devices are typically assembled using modular components manufactured by specialized global suppliers. These components include joints, pylons, liners, sockets, and other mechanical or microprocessor-controlled elements that are configured to meet the needs of individual patients.

**Figure 1:**
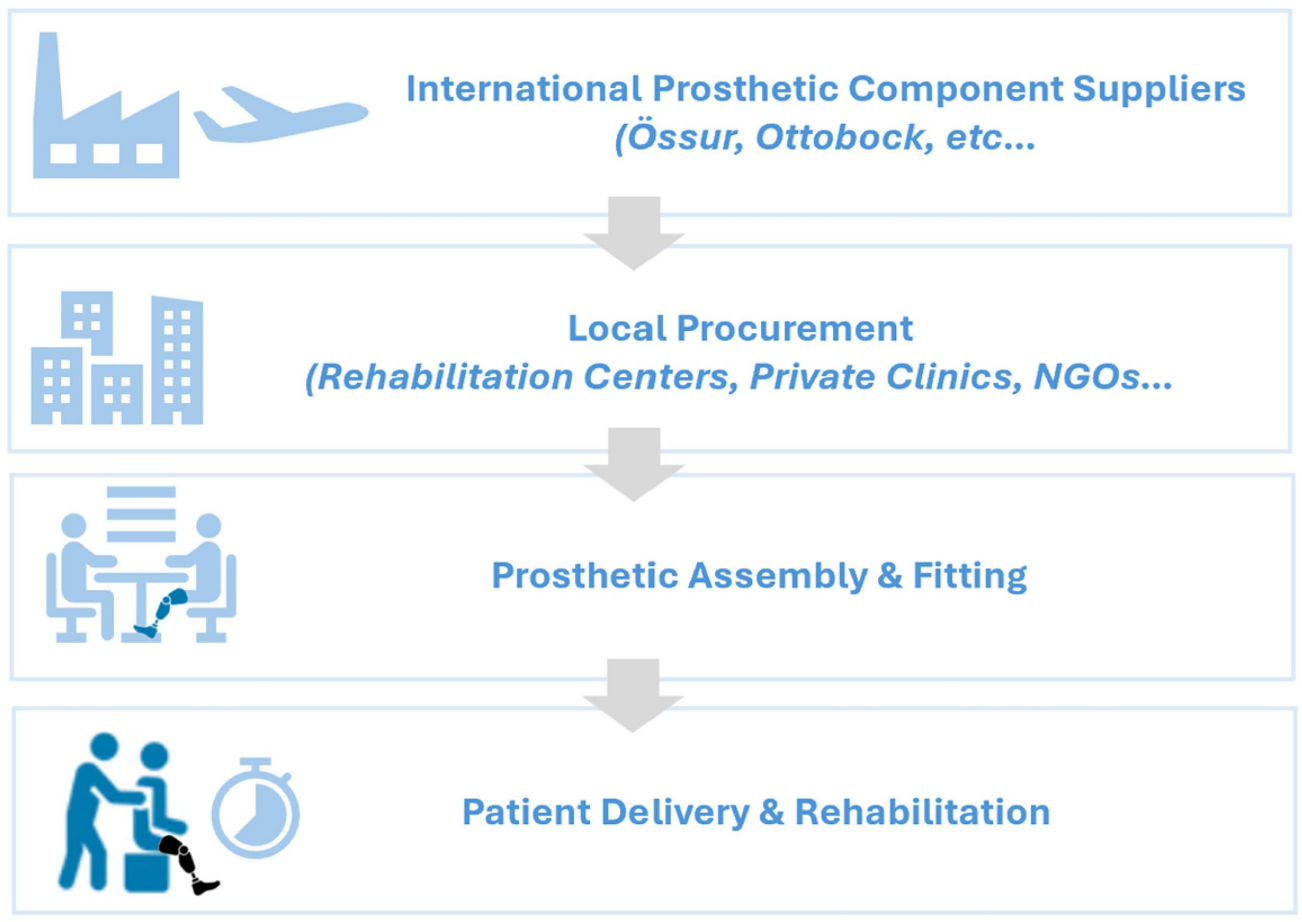
Prosthetics supply chain in Ukraine.

Ukraine's prosthetics sector includes a diverse network of service providers comprising state-owned enterprises, municipal rehabilitation facilities, private providers, which make up the large majority, and non-governmental organizations (17). This mixed provider landscape reflects both the historical development of rehabilitation services and efforts to expand service capacity in response to increasing demand, in which public, private, and non-governmental bodies are governed by different regulations (Figure 1).

In Ukraine, procurement arrangements for prosthetics and assistive technologies vary significantly by institutional form, reflecting differing legal obligations and funding sources. Public sector entities—including ministries, state institutions, and most municipal non-profit enterprises are bound by the Law of Ukraine “On Public Procurement” (No. 922-VIII) (18), and must conduct competitive tenders through the Prozorro e-procurement system, which standardises procedures, enforces transparency, and sets value thresholds for open bidding.

State-owned commercial enterprises (SOEs) are also generally subject to this law when procuring with public funds or operating in regulated sectors, although they may apply more flexible or commercial practices below thresholds or when acting on a purely commercial basis. In contrast, private sector providers of prosthetics are not governed by public procurement law when purchasing inputs and operate under general commercial and civil law frameworks (19, 20).

Non-governmental organisations (NGOs) operate under private law procurement rules and donor-specific regulations (e.g., UN, World Bank), meaning their procurement is typically guided by internal policies emphasising competition and value for money rather than national legislation. As a result, Ukraine's prosthetics market is characterised by a hybrid procurement environment in which public buyers are tightly regulated and transparent, while private and NGO actors retain procedural flexibility, creating both complementarities and fragmentation in market access and purchasing practices. This serves to highlight that there has always been structural, procedural, and decision-making distinctions between public and private purchasing frameworks.

Within this system, individual providers frequently undertake procurement of prosthetic components independently. Providers may purchase components directly from international suppliers or through local distributors. Procurement cycles often occur periodically throughout the year as providers acquire components needed to assemble prosthetic devices for patients.

While decentralized procurement can provide flexibility for providers responding to patient needs, it can also generate structural challenges (21). When procurement occurs through numerous independent purchasing processes, demand for prosthetic components becomes fragmented across multiple institutions (22). This fragmentation may limit opportunities to consolidate purchasing volumes and negotiate favourable pricing with suppliers.

In addition, decentralized procurement arrangements can complicate supply chain coordination. Prosthetic components are frequently sourced from specialized manufacturers operating in international markets (8). Providers may depend on imported components produced by companies such as Össur and Ottobock. When multiple providers procure components independently, suppliers must respond to numerous smaller orders rather than coordinated purchasing volumes across the sector for products that are made to order, not to stock.

The Ukrainian context also highlights the role of digital procurement systems in improving transparency (11). Public procurement processes are supported by the national electronic procurement platform ProZorro, which has been internationally recognized for enhancing transparency and competition in government purchasing. However, not all public rehabilitation service providers in Ukraine use the platform for prosthetics procurement, and there is no obligation for the private sector or NGOs to access public funds. Digital procurement platforms can facilitate open tendering processes and improve visibility of procurement activities across public institutions.

However, while such platforms can improve transparency, they do not automatically address structural fragmentation of demand across multiple buyers (23). When procurement decisions remain decentralized at the level of individual providers, opportunities for coordinated purchasing and supply chain planning may remain limited.

These dynamics illustrate how procurement architecture influences the ability of rehabilitation systems to scale services efficiently. Even when funding is available and service capacity expands, procurement arrangements may affect the reliability of supply chains and the cost structure of prosthetic services.

## 3 Structural challenges in procuring rehabilitation technologies

The procurement of rehabilitation technologies differs in several important respects from procurement of other health products (24). Many procurement systems within the health sector were originally designed to manage high-volume pharmaceutical products and standardized medical supplies. These procurement models often prioritize products purchased in large quantities and distributed through established national supply chains.

Rehabilitation technologies, by contrast, often involve specialized products produced in smaller volumes and tailored to individual patient needs. Prosthetic devices are typically assembled from multiple modular components that must be fitted and adjusted by trained prosthetists. The number of devices produced may vary considerably depending on patient demand and clinical requirements (8).

This structural difference can create challenges within conventional procurement frameworks (25). Procurement procedures optimized for standardized products may not always accommodate the more variable demand patterns associated with prosthetic components and other assistive technologies.

Fragmented procurement can also limit the ability of health systems to benefit from economies of scale (26). When providers procure components individually in relatively small quantities, suppliers may face higher transaction costs associated with processing numerous smaller orders. These costs may be reflected in higher component prices.

Another challenge relates to demand forecasting. Reliable forecasting is critical for ensuring stable supply chains and preventing shortages of essential components. In decentralized procurement environments, data on prosthetic component utilization may be dispersed across multiple institutions. Without integrated data systems, obtaining a comprehensive view of sector-wide demand becomes more difficult (26).

Quality assurance also represents an important consideration (27). Prosthetic components must meet stringent quality standards to ensure safety and reliability for patients. Maintaining consistent quality oversight may be more complex when procurement activities are dispersed across numerous institutions with varying procurement capacities.

These challenges highlight the need for procurement systems that recognize the distinctive characteristics of rehabilitation technologies. Strengthening procurement governance may therefore represent an important opportunity to improve access to assistive products within rehabilitation systems.

## 4 Procurement reform approaches to support rehabilitation systems

Addressing procurement challenges within rehabilitation systems requires approaches that combine efficiency, transparency, and responsiveness to patient needs. Several procurement reform strategies may support improved access to prosthetic technologies and other assistive products.

One approach involves consolidating purchasing demand across providers through pooled procurement mechanisms (22). By aggregating purchasing volumes, pooled procurement can enhance bargaining power with suppliers and enable more favourable pricing arrangements. Consolidated purchasing may also provide suppliers with more predictable demand, improving supply chain stability.

Framework agreements represent another procurement tool with potential relevance for rehabilitation products. Under framework agreements, suppliers are selected through competitive tendering processes and agree to provide products at predetermined pricing over defined time periods. Providers can then purchase components as needed without initiating a full procurement procedure for each transaction. This approach can reduce administrative burdens and enable faster access to specialized components (28).

Improving demand forecasting capabilities is also critical (22). Integrating procurement data systems across rehabilitation providers can help generate more accurate projections of future demand for prosthetic components. Such information can inform procurement planning and enable better coordination between providers and suppliers.

Digital procurement platforms may further enhance transparency and efficiency (11, 23). Electronic procurement systems allow procurement activities to be recorded and monitored in real time, improving accountability and facilitating oversight of public expenditure. Digital systems may also enable aggregation of procurement data across institutions, providing insights into purchasing patterns and market dynamics.

Finally, procurement frameworks incorporating lifecycle costing approaches may support more sustainable financing of rehabilitation technologies. Prosthetic devices often require ongoing maintenance, adjustments, and component replacement, and a thorough assessment of all-inclusive pricing factors such as fitting, maintenance, and service costs (4). Procurement decisions that consider the full lifecycle costs of prosthetic technologies may help ensure that rehabilitation services remain financially sustainable over time.

**Figure 2:**
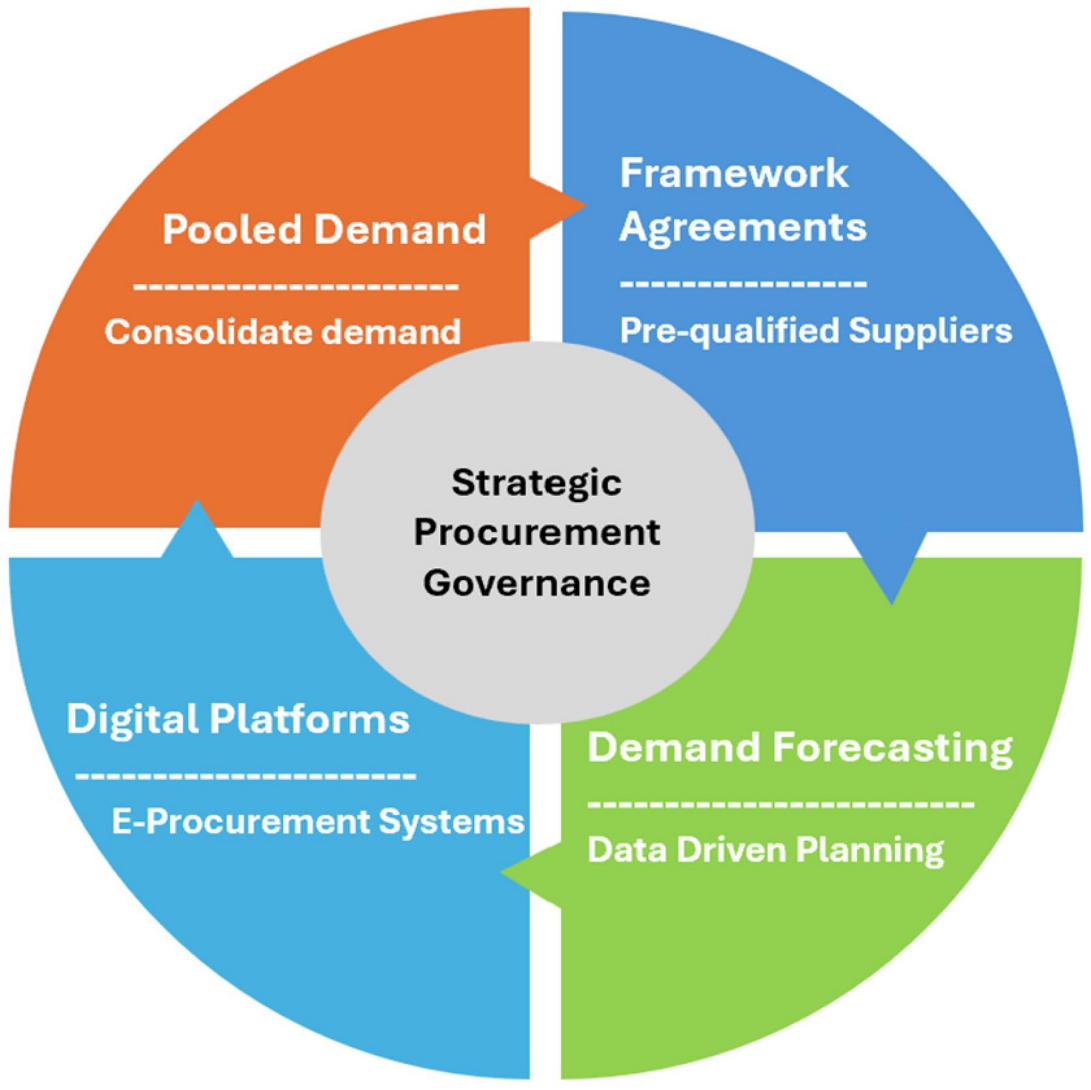
Key elements of effective procurement architecture.

These reform approaches do not eliminate the need for decentralized clinical decision-making by prosthetists and rehabilitation specialists. Rather, they aim to strengthen the institutional systems that support service delivery by improving the efficiency and reliability of procurement processes (Figure 2).

However, it should be noted that while procurement consolidation may generate economies of scale, procurement efficiencies, and improve price transparency, its implementation is not without challenges. Centralising procurement, if not well structured, can reduce flexibility if it consists of a limited number of suppliers to respond rapidly to local needs or supplier disruptions. The transition to a consolidated model may also involve significant administrative effort, including the rationalization of service providers, suppliers, the harmonization of contracts, and the development of new procurement processes. In addition, organizations may incur short-term transition costs related to system changes, staff training, and stakeholder coordination. Care should be given not to over-rely on a smaller range of suppliers and service providers, which could further increase supply risks if key suppliers experience production or logistical difficulties. Consequently, the potential efficiency gains from consolidation should be weighed against these operational and implementation challenges.

## 5 Implications for rehabilitation policy and health systems

The experience of Ukraine's prosthetics sector highlights the importance of considering procurement systems as a structural component of rehabilitation policy (29). While workforce development, infrastructure investment, and clinical standards remain essential elements of rehabilitation system strengthening, procurement governance plays a critical role in enabling these investments to translate into improved access for patients.

Procurement systems shape supplier markets, influence pricing structures, and determine the availability of essential components used in prosthetic devices (24). When procurement systems function effectively, rehabilitation providers can access the materials required for patient care without delays or excessive costs. Conversely, fragmented or poorly coordinated procurement arrangements may constrain service delivery even when funding and clinical capacity expand.

These considerations are particularly relevant in contexts where rehabilitation needs increase rapidly due to conflict, disasters, or demographic change. Health systems responding to such pressures must be able to scale rehabilitation services while maintaining reliable access to assistive technologies. Procurement systems capable of coordinating demand and supporting stable supply chains are therefore essential for system resilience (30).

From a policy perspective, integrating procurement considerations into rehabilitation planning may support more effective health system responses to growing rehabilitation needs. Strategic procurement arrangements can complement investments in workforce development and service infrastructure by ensuring that providers have reliable access to essential components and assistive technologies.

More broadly, strengthening procurement governance aligns with international efforts to expand access to assistive products and rehabilitation services worldwide. Recognizing procurement as a core component of rehabilitation systems may therefore contribute to more effective policy frameworks for improving access to rehabilitation care.

## 6 Conclusion

Procurement architecture represents a critical but often under-recognized determinant of rehabilitation system performance. While procurement processes are frequently viewed as administrative functions, the institutional arrangements through which assistive technologies are purchased directly influence the availability, affordability, and reliability of rehabilitation services.

Ukraine's prosthetics sector provides a contemporary example of how procurement systems shape the ability of health systems to respond to rapidly increasing rehabilitation needs. The experience illustrates how decentralized procurement arrangements, complex supply chains, and fragmented demand patterns may affect the delivery of prosthetic services.

At the same time, the Ukrainian case highlights opportunities for procurement reform to strengthen rehabilitation systems. Approaches such as pooled purchasing, framework agreements, improved demand forecasting, and digital procurement platforms may enhance the efficiency and transparency of procurement processes for assistive technologies.

As countries seek to expand rehabilitation services and improve access to assistive products, procurement governance should be recognized as a foundational element of rehabilitation system development. Strengthening procurement architecture can help ensure that investments in rehabilitation translate into meaningful improvements in mobility, independence, and quality of life for individuals who rely on assistive technologies.
